# Effect of vitamin D supplementation on the severity of stress urinary incontinence in premenopausal women with vitamin D insufficiency: a randomized controlled clinical trial

**DOI:** 10.1186/s12905-022-02024-1

**Published:** 2022-11-04

**Authors:** Sedigheh Khodabandeh Shahraki, Seyedeh Fatemeh Emadi, Mahla Salarfard, Zahra Chenari, Faezeh Tadayyonfar, Maryam Alikamali

**Affiliations:** 1 Department of Community Health Nursing, Razi Faculty of Nursing and Midwifery, Kerman University of Medical Sciences, Kerman, Iran; 2 Department of Midwifery, Ahvaz Jundishapur University of Medical Sciences, Ahvaz, Iran; 3 PhD student in Reproductive Health, Faculty of Nursing and Midwifery, Mashhad University of Medical Sciences, Mashhad, Iran; 4 Department of Reproductive Health, Faculty of Medical Sciences, Tarbiat Modares University, Tehran, Iran; 5 Student Research Committee, Kerman University of Medical Sciences, Kerman, Iran

**Keywords:** Vitamin D3, Stress urinary incontinence, Premenopausal

## Abstract

**Background:**

Urinary incontinence, especially stress urinary incontinence (SUI), is one of the problems experienced by premenopausal women. Given the role of vitamin D in enhancing muscle strength and function, this study explored the effect of vitamin D3 supplementation on SUI in premenopausal women.

**Methods:**

A randomized controlled trial was performed with 60 premenopausal women referring to Kerman gynecological clinic in 2020 and 2021. Eligible women received a 5000-unit vitamin D supplement or placebo weekly for 3 months. The International Consultation on Incontinence Questionnaire-Urinary Incontinence Short Form (ICIQ-SF) was utilized to assess SUI severity before and after the intervention. The t-test, Chi-square test, and repeated measures ANOVA were carried out in SPSS software (version 22) to analyze the data. *P*-values smaller than 0.05 were considered significant.

**Results:**

Before the intervention, there was no significant difference between the intervention and control groups in SUI severity (*P* = 0.652) and the impact of SUI severity on premenopausal women’s lives (*P* = 0.804). In contrast, after 8-12 weeks of vitamin D supplementation, these scores decreased significantly in the intervention group relative to the control group (*P* <  0.001). In addition, after vitamin D supplementation, the number of SUI and urinary leakage symptoms decreased in the intervention group (*P* <  0.001).

**Conclusion:**

Vitamin D supplementation improves SUI in premenopausal women.

**Trial registration:**

This trial was registered with the Iranian Registry of Clinical Trials; https://fa.irct.ir/trial/53474 (IRCT20190724044318N2) on 11/02/2021.

## Introduction

Prior to menopause, a woman goes through perimenopause or premenopause, a period characterized by irregular bleeding and some endocrine disorder symptoms. Aging is associated with a gradual loss of muscle strength [1]. Alongside this, weakened pelvic floor muscles are linked with urinary incontinence, the most frequently reported symptom among pelvic floor disorders [2]. Stress urinary incontinence (SUI) is the most common type of urinary incontinence, with a prevalence of 50% [3]; its prevalence among Iranian women aged 40 to 50 is approximately 38.4% [4]. SUI occurs during times of elevated intraabdominal pressure (e.g., sneezing, coughing, and exercise) [5]. Since levator muscles play an important role in maintaining the urethra’s closure by supporting pelvic organs, strengthening the pelvic floor muscles through exercise and training is recommended as an effective treatment for stress incontinence [6].

It is plausible from a biological standpoint that the epidemic of vitamin D deficiency may have significant clinical effects on the pelvic floor muscles [7]. According to in vivo studies, the vitamin D receptor is located in the bladder neck, and consists of the urothelium and the inner length of the circle, as well as the middle and smooth muscle layers of the outer length of the bladder wall [8]. The presence of vitamin D receptors on both the smooth and skeletal muscles of the bladder, as well as 1-alpha hydroxylase in prostate cells, indicates that vitamin D can aid in both stress and emergency urinary incontinence, which are the most typical types of urinary incontinence in adults [8–10]. It is, therefore, conceivable that vitamin D receptors are dispersed throughout the bladder wall [7].

Epidemiological data regarding the connection between vitamin D status and urinary incontinence are contradictory [11]. According to some previous observational cohort studies, a higher concentration of 25-hydroxyvitamin D has been linked to a lower prevalence and incidence of urinary incontinence [12–15]. Other studies have not found a significant correlation [8–10, 16]. Elin et al. found in a 2011–2014 study examining the effect of vitamin D supplementation on urinary incontinence in older women that the widespread use of moderate vitamin D supplementation did not reduce urinary incontinence [11]. On the other hand, recent studies have found prevalent vitamin D deficiency in women with various types of urinary incontinence, particularly SUI. Indeed, using vitamin D in women to improve urinary incontinence symptoms has produced conflicting results. Midwives can prescribe vitamin D given the low levels of vitamin D in premenopausal women, the higher prevalence of SUI in older women, as well as vitamin D’s safety, economic value, and ease of consumption. Accordingly, we conducted a study to determine the effect of vitamin D supplementation, along with Kegel exercises, on SUI severity in premenopausal women with insufficient vitamin D.

## Methods

### Research setting, design, and participants

This study is designed as a parallel group randomized clinical trial. The protocol of the trial was approved by 82the Ethics Committee of Kerman University of Medical Sciences (ethics code: KMU.REC.1399.555) and registered on the Iranian Registry of Clinical Trials (identifier: IRCT20190724044318N2).

From March 2020 to September 2021, the study enrolled premenopausal women with SUI who were referred to a gynecological clinic affiliated with Kerman University of Medical Sciences.

Prior to sampling, all candidates were assured of the study’s objectives and procedures and were requested to sign informed consent forms as a sign of willingness to participate in the study. Moreover, they were assured they could withdraw from the study anytime.

### Sample size estimation

As per G*Power 3.1 computations, a minimum sample size of *n* = 60 was required, given an effect size of d = 0.8 (based on a pilot study on urinary incontinence severity variable), β = 0.80, α level = 0.05, and 10% attrition.

### Inclusion and exclusion criteria

Literate women aged 40 to 49 years who had serum vitamin D levels below 30 ng/ml(18) and confirmed SUIs were included in the study [8]. Exclusion criteria included being reluctant to continue participation, using hormonal medications, and undergoing urogenital-related surgery. Participants were not included if they had any disorder that interfered with vitamin D absorption, such as inflammatory bowel disease, intestinal bypass surgery, or chronic liver or kidney disease [7], and any neurological disease affecting the urinary system or bowel movements, such as multiple sclerosis, degenerative muscle disease, a history of stroke with spinal cord injury, and chronic or high-grade diabetes [7, 8, 17]. Moreover, all chronic or current infectious diarrhea cases were excluded [7].

### Randomization

The random assignment sequence was generated using a computer program (http://www.random.org) with a 1:1 allocation ratio (blocks 4 and 6).

### Blinding

The one who selected the research subjects, the data collector, the participants, and the data analyst were unaware of the groupings in this experiment (double-blind study). Identical, opaque, consecutively numbered bottles containing codes A (vitamin D capsule) and code B (placebo capsule) were used to conceal the allocation and ensure blinding. The bottles were distributed to the participants in the order in which they entered the study. Someone uninvolved in sampling and evaluating the results prepared the bottles and determined the allocation sequence.

### Intervention program and patient education

In the first session, the researcher educated both the placebo and experimental group members on how to perform the Kegel exercises. The individual lies in a supine position and contracts the pelvic floor muscles for 10 seconds while keeping the legs slightly apart and completely relaxed. The muscles are then released and relaxed for another 10 s. The performer can use her hands to check that the abdominal muscles are relaxed and not tense. The movement is performed 15–20 times and is immediately followed by a posture where the legs are slightly bent/reflexed and tilted toward the abdomen. Alternatively, the exercise can be performed with the knees slightly apart and the arms resting on a table in a set of 15–20 repetitions at rest. The sets can be increased to 25 repetitions when the individual perceives stronger contractions. Alternately, the exercise can be performed with palms and knees on the ground, muscles successively contracted and relaxed for 10 seconds, continuing this sequence for 15–20 repetitions [8]. The Kegel exercise brochure was also distributed to the participants. A weekly telephone check-in ensured that medication was used and Kegel exercises were performed. As the intervention concluded, a urologist re-evaluated the two groups.

SUI was assessed by completing the standard ICIQ-SF (Incontinence Questionnaire - Urinary Incontinence Short) questionnaire at four points: baseline, month 1, month 2, and month 3. This questionnaire includes six questions that examine the SUI condition over the past 4 weeks. Items 1 and 2 pertain to demographic variables, while item 3 concerns urinary incontinence frequency. Item 4 measures leakage volume, while item 5 evaluates SUI’s impact on quality of life.

The scores from items 3, 4, and 5 represent the actual score. Item 6 is an unscored question that specifies the date and type of leakage. ICIQ-SF’s total score may range from 0 to 21 (1–5 mild, 6–12 moderate, 13–18 severe, and 19–21 very severe). Haj Ebrahimi et al. (2012) evaluated the questionnaire’s validity and reliability in the Iranian context. Cronbach’s alpha was 0.75, while the Pearson correlation was 0.93 [16].

Before the participants began to complete the questionnaire, blood samples were taken to measure vitamin D levels. Serum concentrations of 25-hydroxy vitamin D were tested by the LIAISON method in the DIaSorin kits using a Chemiluminescence device. The standard laboratory value was 30–100 ng/ml.

### Statistical methods

T-test and Chi-square tests were utilized to compare the demographic variables of the intervention and control groups using the SPSS 22 statistical software. The paired t-test was used to compare the severity of urinary incontinence and the quality of life of women before and after the intervention in each group. Moreover, the repeated measures ANOVA was employed to compare post-treatment severity of urinary incontinence and life quality between the two groups over time. In addition, the chi-square test was utilized to compare the qualitative variables of urinary incontinence and urinary leakage between the two groups. The significance level of *P* <  0.05 was considered.

## Results

### Characteristics of the patients

**Fig. 1:**
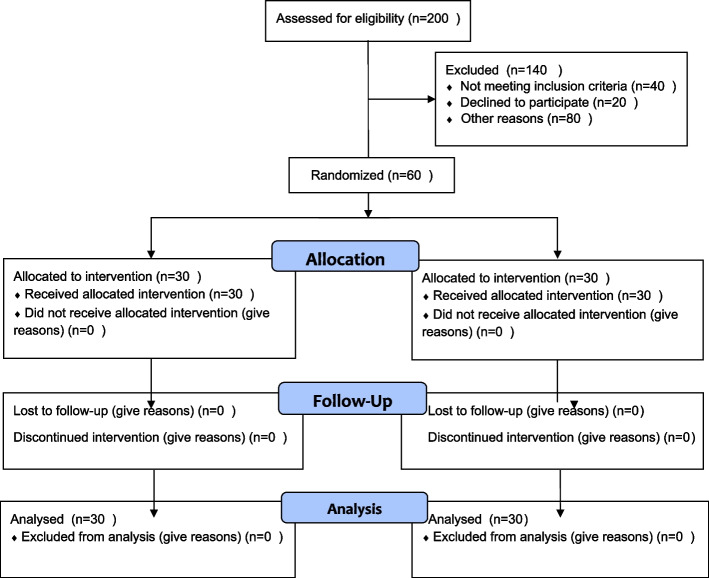
CONSORT flowchart of the participants

We had no attrition in this study due to the good follow-up. Figure 1 presents the patient flowchart. Two hundred patients were initially screened, 120 were deemed ineligible for the research, and 20 refused to participate. Sixty patients were randomized equally into vitamin D or placebo groups (Fig. 1). Of 60 participants, members of the intervention (*n* = 30) and control (*n* = 30) groups completed the questionnaires (Table 1).

**Table 1:** Participants’ baseline characteristics by research group

Variable	Intervention (*n* = 30)	Control (*n* = 30)	*P*-value
Age (years)	44.53 [2.5]	44.23 [2.4]	0.72^a^
Type of delivery			0.5^b^
Natural childbirth	15 (50)	14 (46.7)	
Cesarean section	15 (50)	16 (53.3)	
Number of deliveries	2.9 [1.1]	2.53 [1.1]	0.91^a^
Weight (kg)	68.63 [5.9]	69.46 [5.5]	0.73^a^
Height (cm)	161.56 [8.7]	162.33 [7.7]	0.80^a^
BMI (kg/m^2^)	26.60 [2.5]	26.49 [2.8]	0.72^a^
Serum levels of vitamin D (ng/ml)	25.86 [1.7]	25.73 [2.08]	0.08^a^

The data are expressed as percentages (%) or mean values [standard deviation]

^a^Independent t-test

^b^Chi-square for trend

### Main outcome measures

The mean SUI severities were similar in the groups at baseline (*P* = 0.65) and 4 weeks after the intervention (*P* = 0.66). Nonetheless, at 8 and 12 weeks after the intervention, the vitamin D group’s SUI intensity was significantly lower than that of the placebo group (*P* <  0.0001) (Table 2).

**Table 2:** Comparison of severity of stress urinary incontinence and the impact of the severity of urinary incontinence on the life of premenopausal women in intervention and control groups

Items	Time	Intervention (*n* = 30)	Control (*n* = 30)	*P*-value^a^	Time	Time*Group	Group
Severity of SUI	Before intervention	13.93 ± 3.49	13.53 ± 3.33	0.652	< 0.001	< 0.001	0.001
	Four weeks after intervention	13.13 ± 3.23	13.50 ± 3.30	0.666			
	Eight weeks after intervention	9.16 ± 2.27	12.93 ± 3.69	< 0.001			
	Twelve weeks after intervention	4.66 ± 0.71	12.23 ± 4.21	< 0.001			
	*P*-value^b^	< 0.001	0.010				
The impact of the severity of urinary incontinence on life	before intervention	7.90 ± 2.09	7.76 ± 2.06	0.804	< 0.001	< 0.001	< 0.001
	4 weeks after intervention	7.36 ± 1.93	7.76 ± 2.06	0.442			
	8 weeks after intervention	5.06 ± 1.28	7.40 ± 2.29	< 0.001			
	12 weeks after intervention	2.23 ± 0.62	6.93 ± 2.79	< 0.001			
	*P*-value^b^	< 0.001	0.009				

Data are expressed as mean ± standard deviation

^a^independent *t*-test between groups

^b^repeated measures analysis of variance (Bonferroni correction) within groups

Before the intervention, SUI severity (*P* = 0.652) and SUI’s impact on quality of life (*P* = 0.804) did not differ significantly between the study groups (Table 2). In addition, despite a reduction in SUI severity (*P* = 0.666) and an improvement in SUI’s impact on life (*P* = 0.442) in the intervention group, no significant difference was observed between the intervention group and the control group 4 weeks after the intervention. Eight and 12 weeks after the intervention, there was a significant difference between the two groups regarding a decline in SUI severity and improvement in life quality in the intervention group compared with the control group. More specifically, a significant difference was observed four, eight, and twelve weeks after the intervention compared to before the intervention (Table 3). In contrast, the mean changes over time were insignificant in the control group. In addition, repeated-measures ANOVA revealed that the change in SUI severity scores and the improvement in SUI’s impact on life in both groups decreased significantly over time (*P* <  0.01).

**Table 3:** Mean changes in stress urinary incontinence severity and the impact of the severity of urinary incontinence on life in control and intervention groups

Variables	time	Intervention		Control	
		Mean different	*P*	Mean different	P^*^
Severity of SUI	Before-4 weeks	0.800	< 0.001	0.03	1
	Before-8 weeks	4.76	< 0.001	0.60	0.259
	Before-12 weeks	9.26	< 0.001	1.30	0.084
	4 weeks–8 weeks	3.96	< 0.001	0.56	0.153
	4 weeks–12 weeks	8.46	< 0.001	1.26	0.063
	8 weeks–12 weeks	4.50	< 0.001	0.70	0.095
The impact of the severity of urinary incontinence on life	Before-4 weeks	0.53	< 0.001	0	1
	Before-8 weeks	2.83	< 0.001	0.36	0.402
	Before-12 weeks	5.66	< 0.001	0.83	0.056
	4 weeks–8 weeks	2.30	< 0.001	0.36	0.265
	4 weeks–12 weeks	5.13	< 0.001	0.833	0.053
	8 weeks–12 weeks	2.83	< 0.001	0.46	0.066

^*^Benferroni correction

Table 2 displays that the effect of measurement time on the severity of SUI and the amelioration of SUI’s impact on life in premenopausal women was statistically significant (*P* <  0.001). Therefore, there was a significant difference in the average severity of SUI and the improvement of SUI’s impact on life at baseline, 4 weeks, 8 weeks, and 12 weeks after the intervention. Furthermore, the interaction effect of time and group was significant (*P* <  0.001). Indeed, there was a statistically significant difference between the average severity of SUI and the improvement of SUI’s impact on the lives of premenopausal women in the intervention and control groups at different times (*P* <  0.001).

As illustrated in Table 2, the effect of the group on SUI severity and improvement in SUI’s impact on life in premenopausal women was statistically significant (*P* <  0.001). More specifically, there was a significant difference between the mean scores of SUI severity and the improvement of SUI’s impact on life among premenopausal women in the intervention and control groups.

Table 4 depicts the frequency and relative percentage of urinary incontinence and urine leakage between premenopausal women in four stages as per the study groups.

**Table 4:** The frequency and relative percentage of urinary incontinence and urine leakage in premenopausal women in four stages by study groups

Item	Severity of symptoms	before intervention		4 weeks after		8 weeks after		12 weeks after	
		Intervention	Control	Intervention	Control	Intervention	Control	Intervention	Control
*Prevalence of incontinence*	1 weekly	–	–	–	–	1 (3.3)	0 (0)	17 (56.7)	0 (0)
	2 or 3 weekly	3 (10)	4 (13.3)	4(13.3)	4 (13.3)	14 (46.7)	6 (20)	13 (43.3)	7 (23.3)
	1 daily	9 (30)	11 (36.7)	10(33.3)	11 (36.7)	12 (40)	10 (33.3)	0 (0)	11 (36.7)
	Several daily	11 (36.7)	11 (36.7)	12(40)	12 (40)	3 (10)	11 (36.7)	0 (0)	11 (36.7)
	Always	7 (23.3)	4 (13.3)	4(13.3)	3 (10)	0 (0)	3 (10)	0 (0)	1 (3.3)
*P*-value ^a^		0.762		0.979		0.018		< 0.001	
Leakage rate	Low	3 (10)	2 (6.7)	4 (13.3)	2 (6.7)	14 (46.7)	4 (13.3)	30 (100)	6 (20)
	Medium	15 (50)	18 (60)	15 (50)	18 (60)	16 (53.3)	17 (56.7)	0 (0)	15 (50)
	Hight	12 (40)	10 (33.3)	11 (36.7)	10 (33.3)	0 (0)	9 (30)	0 (0)	9 (30)
*P*-value^a^		0.721		0.610		0.001		< 0.001	

^a^Chi-square test

## Discussion

Vitamin D supplementation significantly improved SUI severity after 3 months of intervention in the experimental group compared to the placebo group. Pelvic floor disorders are widespread, affecting one in every three women as they age [18]. Pelvic floor muscles are largely responsible for pelvic organ support and urinary retention. Weakness of these muscles is linked with urinary incontinence, the most frequently reported symptom of pelvic floor disorders [2]. Urinary incontinence affects more than 28% of the population, with SUI being the most prevalent type [19]. High body mass index (BMI) and pelvic organ prolapse [20] were found to have a strong correlation with urinary incontinence [21]. The mean BMI in our study was 26.5 (2.6), which is in the overweight range and a contributor to women’s urinary incontinence.

Although this type of incontinence increases with age, the condition does not merely affect the elderly; it also affects 30% of middle-aged women [22]. Vitamin D levels decline with age. Age also decreases calcium absorption, which corresponds to a decline in vitamin D. The mean age in this study was 44.38 ± 2.45 years. Among micronutrients, vitamin D is known to have a pandemic deficiency [23]. Vitamin D receptors are found in the bladder and the pelvic floor muscles’ striated muscles [8, 10]. While vitamin D deficiency exhibits the most apparent impact on musculoskeletal health [5], it is one of the possible causes of urinary incontinence in older women [24].

In a study conducted by Vaughan et al. [13], preliminary findings indicated a linkage between vitamin D and the prevalence of urinary incontinence in older individuals living in the community, which is consistent with the present study’s findings.

Sharma et al. [5] studied vitamin D concentrations in women with SUI at a tertiary referral center in India, finding a significantly high rate of vitamin D deficiency in these women. Given the vitamin D shortage, vitamin D supplementation was employed in this investigation to determine the resultant effect, which is consistent with the present study’s findings.

Stafne et al. [2] studied the effect of vitamin D on SUI in pregnant women, indicating that serum vitamin D concentrations below 50 nmol/l were associated with an increased risk of incontinence, especially SUI. Consistent with our findings, this study confirmed the association between vitamin D and SUI, even in pregnant women.

These findings were compatible because vitamin D has receptors in musculoskeletal tissue that affect muscle strength and function. Indeed, it can be beneficial in alleviating SUI by strengthening the detrusor muscles of the bladder and pelvic floor muscles.

Markland et al. [24] investigated vitamin D consumption and the 10-year risk of urinary incontinence in elderly and middle-aged women. According to the authors, there was a link between moderate vitamin D consumption and the incidence of urinary incontinence in middle-aged women. Contrary to our study, the women in Markland et al.’s study did not have urinary incontinence at the start, and the serum level of vitamin D was not measured. Therefore, there may not be a correlation between these cases and urinary incontinence. This must be investigated. Alongside this, another study (2021) found that supplementation with moderate doses of vitamin D did not reduce urinary incontinence in older women [11]. This contradiction in results may be due to differences in the age range of the participants. Furthermore, as demonstrated by Kamronrithisorn et al., the effects of vitamin D supplementation may depend on patients’ baseline vitamin D levels, type of vitamin D supplements, and duration of supplementation [25].

On the other hand, excessive vitamin D can increase blood calcium levels, potentially resulting in a condition known as hypercalcemia. Fatigue, loss of appetite, weight loss, excessive thirst, excessive urination, dehydration, constipation, irritability, nervousness, ringing in the ear (tinnitus), muscle weakness, nausea, vomiting, dizziness, confusion, disorientation, high blood pressure, and heart arrhythmias are some of the symptoms. Long-term complications of untreated hypervitaminosis D include kidney damage, kidney stones, kidney failure, excessive bone loss, and calcification of arteries and soft tissues. In addition, high blood calcium levels can lead to abnormal heart rhythms [26].

Some strengths of this study include its random sample allocation, double-blindness, and no participant attrition. Vitamin D serum levels were measured at baseline, and women with insufficient vitamin D serum levels were enrolled in the study and given vitamin D supplements to prevent drug overdose. Both groups were taught Kegel exercises as part of the intervention. However, the study was limited in certain respects. It did not mention the individuals’ diets in terms of vitamin D intake through daily food and did not follow up with them after vitamin D3 supplementation ceased. We did not consider the serum vitamin D levels of the participants again after the study’s conclusion. Also, at the beginning of the study, the participants were not separated based on the severity of their vitamin D deficiency. Indeed, each participant received a single dose of the supplement, regardless of the severity of their vitamin D deficiency, whereas the severity of this deficiency can affect the severity of incontinence. The research population consisted exclusively of women. Hence, the findings cannot be generalized to men. As a result, one cannot judge the consequences of drug withdrawal. Further research may be conducted with larger sample sizes.

## Conclusion

In premenopausal women, vitamin D supplementation alleviated the severity of SUI. Hence, vitamin D supplements are recommended for premenopausal women to improve their SUI because they are readily available, cost-effective, and have no harmful effects.

## Data Availability

The data are available upon request to the corresponding author after signing appropriate documents in line with ethical application and the decision of the Ethics Committee.

## Abbreviations

SUI: Stress urinary incontinence
ICIQ-SF: Incontinence Questionnaire -Urinary Incontinence Short
RCT: Randomized controlled trial
